# Cardiorenal Risk Profiles Among Data-Driven Type 2 Diabetes Sub-Phenotypes: A *Post-Hoc* Analysis of the China Health and Nutrition Survey

**DOI:** 10.3389/fendo.2022.828403

**Published:** 2022-04-06

**Authors:** Hui Gao, Kan Wang, Wensui Zhao, Jianlin Zhuang, Yu Jiang, Lei Zhang, Qingping Liu, Fariba Ahmadizar

**Affiliations:** ^1^Changning Center for Disease Control and Prevention, Shanghai, China; ^2^Department of Epidemiology, Erasmus Medical Center, Rotterdam, Netherlands; ^3^PuDong New Area Center for Disease Control and Prevention, Shanghai, China; ^4^Julius Global Health, University Utrecht Medical Center, Utrecht, Netherlands

**Keywords:** type 2 diabetes, cardiovascular risk, renal function, cluster analysis, trajectory analysis

## Abstract

**Background and Aim:**

Evidence about recently proposed data-driven clusters of type 2 diabetes (T2D) is mainly about its prognostic effects and Western populations. We tested the applicability of this clustering approach among the Chinese population. We further investigated the cardiorenal risk profiles among different T2D sub-phenotypes cross-sectionally and before diabetes diagnosis.

**Methods:**

With the use of data from the China Health and Nutrition Survey (1989–2009), 6,728 participants with available fasting blood samples and completed questionnaires in the 2009 survey were included. Glycemic statuses (normoglycemia, prediabetes, and new-onset T2D) were defined according to the 2020 American Diabetes Association criteria. Data-driven cluster analysis was conducted among new-onset T2D based on five variables: age at onset, body mass index (BMI), hemoglobin A1c, homeostasis model estimates of β-cell function, and insulin resistance. Linear regression models were used to cross-sectionally examine the differences of cardiorenal risk factors (body fat distribution, blood pressure, lipid profiles, and kidney function) between glycemic statuses. Mixed-effects models were used to explore a maximum of 20-year trajectories of cardiovascular risk factors (body fat distribution and blood pressure) before diabetes diagnosis.

**Results:**

Among 557 (8.3%) new-onset T2D, four sub-phenotypes were found, with 57 (10.2%) assigned to the severe insulin-resistant diabetes (SIRD), 72 (12.9%) to the severe insulin-deficient diabetes (SIDD), 167 (30.0%) to the mild obesity-related diabetes (MOD), and 261 (46.9%) to the mild age-related diabetes (MARD). People clustered within different T2D sub-phenotypes had different cardiorenal risk profiles. Three T2D sub-phenotypes (SIRD, SIDD, and MOD) had worse cardiorenal abnormalities, while the risk burden in the MARD sub-phenotype was similar to that in prediabetes. Compared with people with other T2D sub-phenotypes, people in the MOD sub-phenotype had a faster increment in BMI, waist, upper arm circumference, and triceps skinfold up to 10 years before diagnosis. Blood pressure was less distinct in different T2D sub-phenotypes; however, SIDD and MOD clusters had higher blood pressure levels before diabetes diagnosis.

**Conclusions:**

Data-driven T2D sub-phenotyping is applicable in the Chinese population. Certain sub-phenotypes such as MARD only have a minor cardiorenal risk burden, and distinct cardiovascular risk development occurs long before diabetes diagnosis. Our findings can help improve early prevention and targeted treatment for diabetes.

## Introduction

Type 2 diabetes (T2D), comprising over 90% of all people with diabetes in China, is a complex metabolic disorder characterized by insulin resistance and relative insulin deficiency (1). Since T2D is a heterogeneous phenotype, failure in classification followed by the lack of tailored strategies might be responsible for the poor control of diabetes complications, such as macro- and microvascular disease (2–4). Recently, Ahlqvist and colleagues have challenged the current paradigm of adult-onset diabetes sub-phenotyping using data-driven cluster analysis. Based on six variables—glutamate decarboxylase antibodies, age at diagnosis, body mass index (BMI), hemoglobin A1c (HbA1c), and homeostasis model estimates of β-cell function (HOMA-β) and insulin resistance (HOMA-IR)—they stratified people with T2D into five subgroups with different clinical features and risks for developing diabetic complications (5). Several subsequently published studies have further examined the generalizability (6–8) and clinical utility (9) of the Ahlqvist study. Different T2D sub-phenotypes with various degrees of whole-body and adipose-tissue insulin resistance result in a different prevalence of diabetes complications such as early stages of non-alcoholic fatty liver disease and diabetic neuropathy (8). This T2D sub-phenotyping eventually could help improve targeted prevention and treatment. However, there is still limited evidence about the subclinical cardiorenal risk profiles, such as blood pressure, lipid profiles, and kidney function, and there is no evidence about the changes of these risk factors before the diabetes diagnosis, which might preclude optimal timing for early prevention.

In addition, the current literature mainly focuses on populations of Caucasian backgrounds or from Western countries. It is unclear whether T2D sub-phenotypes in China are similar to those reported in the literature (10). Furthermore, the characteristics and risk profile of these distinct sub-phenotypes in a Chinese population are unknown. The prevalence of T2D has increased dramatically in China from 0.67% in 1980 to 11.2% in 2017 (11). Considering the importance of ethnic differences in diabetes diagnosis and prognosis (12–14), studies on the Western population may be inappropriate to help develop stratified treatment strategies for Chinese people with diabetes.

Therefore, in this study, we used data from the prospective China Health and Nutrition Survey (CHNS) to test whether T2D sub-phenotypes can be generalized to the Chinese population using the same approach. We investigated the cardiorenal risk profiles (body fat distribution, blood pressure, lipid profiles, and kidney function) within different glycemic statuses (normoglycemia, prediabetes, and data-driven T2D sub-phenotypes). We further explored trajectories of cardiovascular risk factors preceding the diabetes diagnosis.

## Material and Methods

### Study Design and Population

The CHNS is an ongoing nationwide cohort designed to investigate health and nutritional status among Chinese populations. The detailed study design has been described elsewhere (15). Briefly, the study sample was drawn using a multistage, random cluster process from about 7,200 households in 15 provinces and municipal cities in China. Ten rounds were completed between 1989 and 2015.

Overnight fasting blood samples were collected in 2009. Thus, this survey was used for our analyses to classify different glycemic statuses and explore the trajectories of cardiovascular risk factors before the diabetes diagnosis, retrospectively. An overview of the study population is shown in **Figure S1**. Briefly, among the participants enrolled in the 2009 CHNS survey (n = 18,816), the following were excluded: those who were pregnant, were without an available blood sample, had a history of diabetes (n = 9,669), were younger than 18 years (n = 854), and had missing variables for the cluster analysis (n = 41). Those with missing outcomes of interest, cardiorenal risk factors (n = 1,398) or covariates (n = 126), were further excluded; 6,728 participants were included in our analyses.

### Measurements

Information about demographic, lifestyle, and medical history was obtained through structured questionnaires administered by trained field workers in the 2009 survey. Education level was classified as illiterate (no formal education), primary school, middle school, and high school or higher. Current smoking was defined as having a positive answer to the question, “Do you still smoke cigarettes or a pipe?” Alcohol consumption was assessed using the question, “During the past year, have you drank beer or any other alcoholic beverage? If yes, how often do you drink?” and stratified into no alcohol consumption, 1–2 times per week or less, and 3–4 times per week or more (16). Physical activity level was measured by time per week the participant spent doing moderate or vigorous activities, including martial, gymnastic, track and field, soccer, and basketball. Insufficient physical activity was defined as having less than 150 min of moderate or 75 min of vigorous activities per week (17). Use of blood pressure- or glucose-lowering medications and self-reported doctor-diagnosed cardiopulmonary disease (myocardial infarction, stroke, and asthma) were also collected.

Fasting blood glucose (FBG) was tested using the glucose oxidase method (Hitachi 7600; Randox, Crumlin, UK). HbA1c was measured from whole blood using HLC/HPLC method (HLC-723G7, Tosoh, Tokyo, Japan). Insulin was determined using the radioimmunology method (Gamma counter XH-6020; North Institute of Biological Technology, Beijing, China). Serum creatinine and lipid profiles including triglyceride, total cholesterol, high-density lipoprotein-cholesterol (HDL-C), low-density lipoprotein-cholesterol (LDL-C), Apolipoprotein A-1 (Apo-A), Apolipoprotein B (Apo-B), and lipoprotein-a (Lp(a)) were assessed according to routine clinical protocols (Hitachi 7600; Randox, UK). The homeostasis model assessment was used to estimate insulin resistance (HOMA-IR) and β-cell function (HOMA-β), and the 2009 Chronic Kidney Disease Epidemiology Collaboration equation (CKD-EPI) (18) was used to calculate the estimated glomerular filtration rate (eGFR).

Information about anthropometric (height, weight, waist–hip ratio, upper arm circumference, and triceps skinfold) and blood pressure were obtained through detailed physical examination in each survey, except for waist–hip ratio beginning in 1993. Anthropometric data were measured by trained healthcare workers following standardized protocols and described in detail elsewhere (19, 20). Briefly, height was measured to the nearest 0.1 cm without wearing shoes using a portable stadiometer. Weight was measured to the nearest 0.1 kg using a calibrated beam scale while wearing lightweight clothing. Waist circumference was measured at a point midway between the lowest rib and the iliac crest in a horizontal plane using non-elastic tape. Hip circumference was measured with the same tape to the nearest 0.1 cm at the maximum circumference over the buttocks. Upper arm circumference was measured to the nearest 0.1 cm with the left arm hanging relaxed. The measurement was taken midway between the tip of the acromion and the olecranon process. Triceps skinfold was measured to the nearest mm in triplicate with a Lange skinfold caliper (Beta Technology Ltd., Cambridge, MA, USA) having a pressure of 10 g/mm^2^ of contact surface area. The measurement was taken over the triceps muscle at the midpoint of the left posterior upper arm.

### Ascertainment of Prediabetes and Type 2 Diabetes

Glycemic statuses (normoglycemia, prediabetes, and newly diagnosed T2D) were defined based on the 2020 diagnostic criteria from the American Diabetes Association (21). New-onset T2D was defined as having FBG ≥ 7.0 mmol/L (126 mg/dl) or HbA1c ≥6.5% (48 mmol/mol). Non-diabetic participants were further classified into prediabetes [defined as 5.6 mmol/L (100 mg/dl) ≤ FBG ≤ 6.9 mmol/L (125 mg/dl) or 5.7% (39 mmol/mol) ≤ HbA1c ≤ 6.4% (47 mmol/mol)] and normoglycemia [defined as having FBG < 5.6 mmol/L (100 mg/dl) and HbA1c < 5.7% (39 mmol/mol)].

### Statistical Analysis

#### Cluster Analysis

The cluster analysis was conducted among the new-onset T2D subgroups. Since the glutamate decarboxylase antibody data are unavailable in the CHNS survey, age at diagnosis, BMI, HbA1c, HOMA-IR, and HOMA-β as clustering variables were included. All these selected variables were centered and standardized, and those with extreme outliers (>5 SDs from the mean) were excluded. K-means clustering with a k value of four was performed afterward. Cluster stability was assessed based on the cluster distribution difference by running the K-means algorithm 30 times (5, 6).

#### Cross-Sectional Analysis

Multivariate linear regression models were used to examine the differences of cardiorenal risk factors (BMI, waist, waist–hip ratio, upper arm circumference, triceps skinfold, systolic and diastolic blood pressure, lipid profiles, and kidney function) between different glycemic statuses (normoglycemia, prediabetes, and four diabetes sub-phenotypes) in the 2009 survey. Outcomes were firstly standardized to allow for direct comparisons of effect sizes. Two models were fitted: crude model adjusted for age, sex, education, residence, and marital status; and full model additionally adjusted for smoke status, alcohol consumption, sleep, exercise status, sedentary behavior, use of blood pressure-lowering medication, and prevalent cardiopulmonary disease. For blood pressure outcomes, the use of blood pressure-lowering medication was also included in the crude model.

#### Trajectory Analysis

The trajectories of cardiovascular risk factors were explored before diagnosing different glycemic statuses using mixed-effects models (22). The observation period for those retrospective trajectories started at the 2009 survey date (year 0). Trajectories of the following outcomes were followed back in time to the first available examination: BMI, waist, waist–hip ratio, upper arm circumference, triceps skinfold, and systolic and diastolic blood pressure. Mixed-effects models could use all available data and account for the within-person correlation. Time dependence was allowed to vary across subgroups; quadratic and cubic terms for time were included in the subgroups, when significant. For normoglycemic individuals, year 0 was merely a time point in a normal life course, and linear models were fitted. The trajectory analysis was adjusted for age at diagnosis and sex. Pairwise differences in growth curves between subgroups were checked using the F-tests. To account for multiple testing comparing 15 pairs of glycemic statuses, the Bonferroni-adjusted significance level of 0.05/15 = 0.003 was used for the F-tests for each cardiovascular risk factor.

Information on covariables was missing for only 1.8%; therefore, no imputation was used. Data were handled and analyzed with SPSS Statistics version 25.0.0.1 (IBM Corp., Armonk, NY, USA) and R, CRAN version 4.0.5. All analyses were performed at the significance level of 0.05 (2-tailed), unless specified.

## Results

### Characteristics of People With Different Type 2 Diabetes Sub-Phenotypes

**Figure 1:**
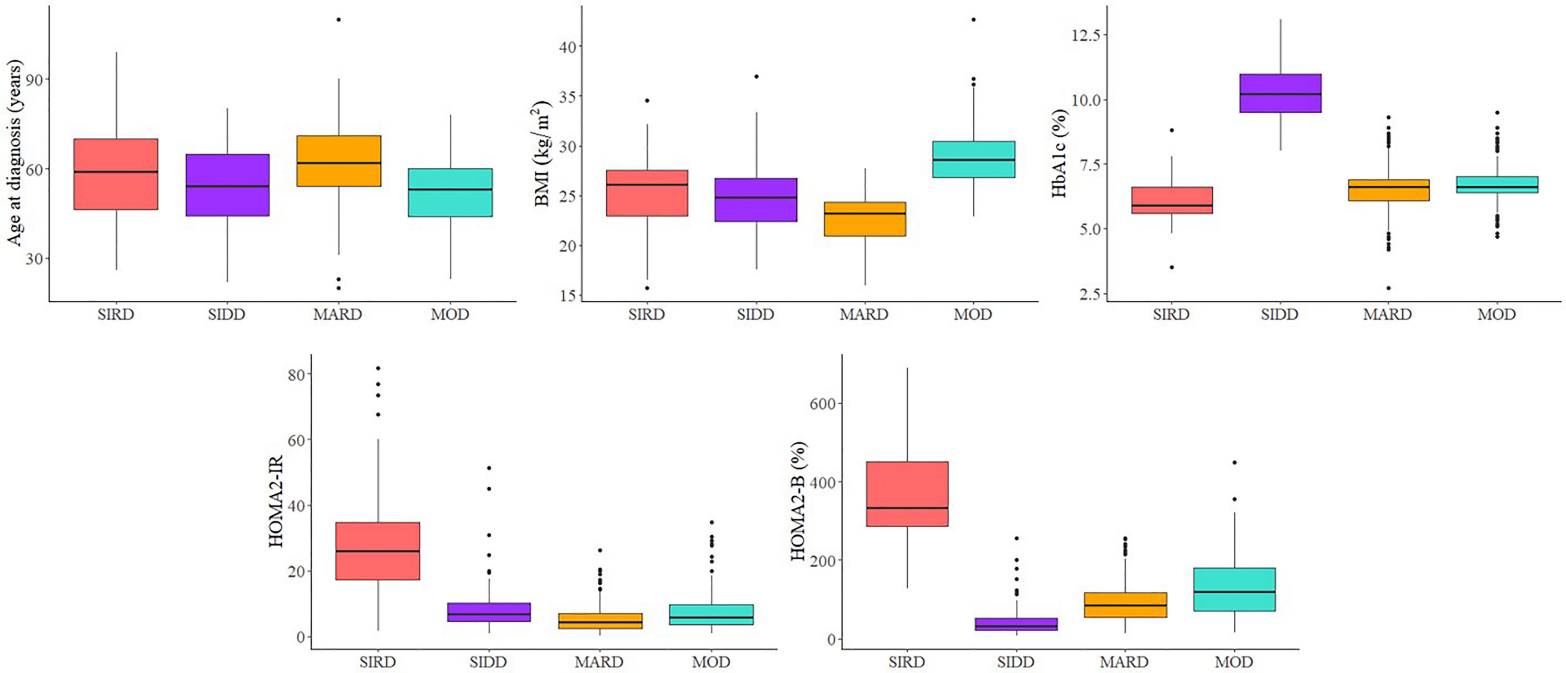
Type 2 diabetes sub-phenotypes characteristics. SIRD, severe insulin-resistant diabetes; SIDD, severe insulin-deficient diabetes; MARD, mild age-related diabetes; MOD, mild obesity-related diabetes.

Of the 6,728 adult participants, 3,436 (51.1%) were normoglycemia, 2,735 (40.7%) had prediabetes, and 557 (8.3%) had new-onset T2D. As shown in **Figure 1** and **Table S1**, four sub-phenotypes among the new-onset diabetes were identified; 57 (10.2%) out of 557 patients were assigned to severe insulin-resistant diabetes (SIRD) sub-phenotype. Patients with SIRD were characterized by insulin resistance (high HOMA-IR) and relatively high BMI. A total of 72 (12.9%) of 557 patients were assigned to the severe insulin-deficient diabetes (SIDD) sub-phenotype, which is characterized by poor glycemic control (high HbA1c) and low insulin secretion (low HOMA-β). A total of 167 (30.0%) of 557 patients, mainly characterized by obesity (high BMI), were assigned to the mild obesity-related diabetes (MOD) sub-phenotype. A total of 261 (46.9%) of 557 patients were assigned to the mild age-related diabetes (MARD) sub-phenotype, who were older at onset age and had minor metabolic abnormalities.

### Cardiorenal Risk Profiles Among Different Glycemic Statuses

**Figure 2:**
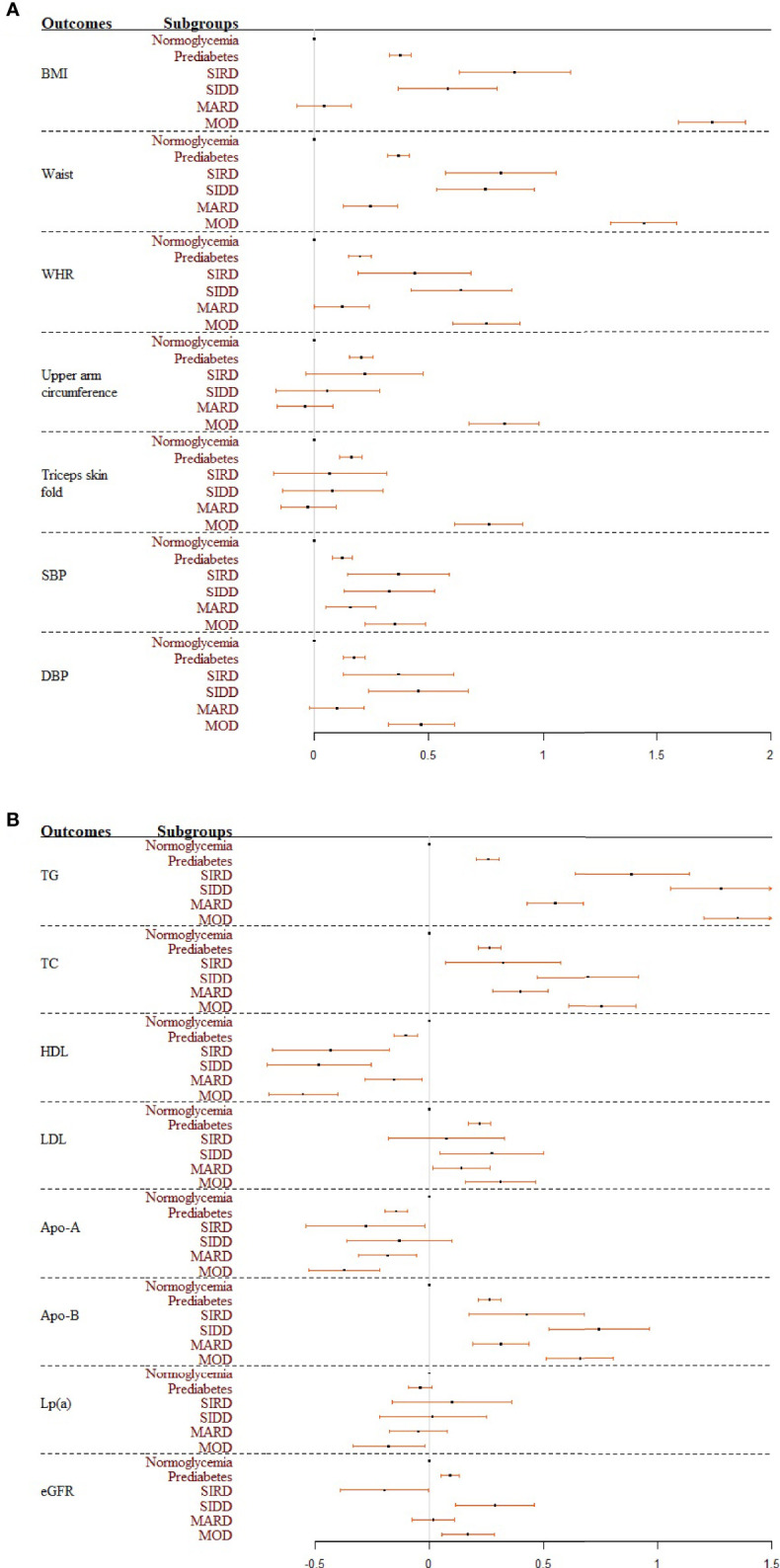
**(A, B)** Forest plots summarizing the coefficients and 95% confident intervals of standardized cardiovascular risk factors among glycemic statuses. Results were based on the full model. SIRD, severe insulin-resistant diabetes; SIDD, severe insulin-deficient diabetes; MARD, mild age-related diabetes; MOD, mild obesity-related diabetes.

**Table 1** shows characteristics of covariates and cardiorenal risk factors at the time of diabetes diagnosis among different glycemic statuses (normoglycemia, prediabetes, and four T2D sub-phenotypes). Results from multivariate linear regression models showed that patients with MOD had the highest levels of BMI, waist circumference, upper arm circumference, and triceps skinfold (**Figure 2A**). Specifically, in the full models and compared to individuals with normoglycemia, those in other subgroups had a higher level of the standardized waist circumference, with mean difference (95% CI) 0.37 (0.32, 0.41) in prediabetes, 0.25 (0.13, 0.36) in MARD, 0.75 (0.53, 0.96) in SIDD, 0.82 (0.57, 1.06) in SIRD, and 1.44 (1.30, 1.59) in MOD (**Table S5**). By comparing the effect sizes among glycemic statuses and body fat distribution indices, we found that compared to BMI, waist circumference showed similar distribution and effect sizes among T2D sub-phenotypes. For blood pressure, compared to normoglycemia, all four T2D sub-phenotypes had higher blood pressure levels. Among them, MARD had the lowest effect size, comparable to prediabetes (**Figure 2A**).

**Table 1:** Characteristics of the study participants at the time of diagnosis among different glycemic statuses.

		Normoglycemia (n = 3,436)	Prediabetes (n = 2,735)	SIRD (n = 57)	SIDD (n = 72)	MARD (n = 261)	MOD (n = 167)
Age, years		47.0 (15.1)	54.2 (13.7)	58.4 (14.6)	54.7 (14.9)	62.1 (11.3)	53.2 (11.7)
Sex, female		1,876 (55%)	1,458 (53%)	28 (49%)	31 (43%)	141 (54%)	82 (49%)
Education level							
	Illiterate	698 (20%)	764 (28%)	16 (28%)	17 (24%)	100 (38%)	43 (26%)
	Primary school	663 (19%)	520 (19%)	16 (28%)	18 (25%)	58 (22%)	40 (24%)
	Middle school	1,220 (36%)	865 (32%)	17 (30%)	26 (36%)	55 (21%)	53 (32%)
	High school or higher	855 (25%)	586 (21%)	8 (14%)	11 (15%)	48 (18%)	31 (19%)
Residence							
	Urban	1,103 (32%)	808 (30%)	16 (28%)	25 (35%)	91 (35%)	53 (32%)
	Rural	2,333 (68%)	1,927 (70%)	41 (72%)	47 (65%)	170 (65%)	114 (68%)
Married		2,838 (83%)	2,361 (86%)	47 (82%)	64 (89%)	219 (84%)	150 (90%)
Current smoker		937 (27%)	773 (28%)	20 (35%)	18 (25%)	74 (28%)	51 (31%)
Frequency of alcohol consumption							
	No alcohol consumption	2,326 (68%)	1,835 (67%)	39 (68%)	46 (64%)	172 (66%)	99 (59%)
	1–2 times/week or less	695 (20%)	489 (18%)	9 (16%)	17 (24%)	41 (16%)	29 (17%)
	3–4 times/week or more	415 (12%)	411 (15%)	9 (16%)	9 (13%)	48 (18%)	39 (23%)
Sleep, hours per day		7.8 (2.2)	7.8 (1.9)	8.4 (1.4)	7.8 (2.6)	7.7 (1.7)	7.9 (1.9)
Insufficient physical activity		3,320 (97%)	2,650 (97%)	56 (98%)	71 (99%)	258 (99%)	163 (98%)
Sedentary behavior, hours per day		0.8 (0.6)	0.8 (0.6)	0.7 (0.4)	0.7 (0.4)	0.8 (0.6)	0.7 (0.5)
Prevalent myocardial infarction		14 (0.4%)	29 (1%)	0 (0%)	2 (3%)	3 (1%)	1 (1%)
Prevalent stroke		26 (1%)	46 (2%)	4 (7%)	1 (1%)	8 (3%)	2 (1%)
Prevalent asthma		26 (1%)	43 (2%)	3 (5%)	1 (1%)	7 (3%)	2 (1%)
**Body fat distribution**							
	Height, cm	160.6 (8.6)	160.5 (8.6)	159.8 (8.9)	162.4 (9.1)	159.8 (8.1)	161.0 (8.2)
	Weight, kg	58.3 (10.5)	61.8 (11.1)	65.2 (11.3)	65.7 (12.0)	58.3 (8.9)	74.6 (9.2)
	Body mass index, kg/m^2^	22.5 (3.2)	23.9 (3.4)	25.5 (3.9)	24.8 (3.4)	22.8 (2.4)	28.8 (2.8)
	Waist circumference, cm	79.8 (9.6)	84.6 (9.8)	89.6 (10.5)	89.3 (9.5)	84.2 (8.8)	96.1 (8.8)
	Hip circumference, cm	92.7 (7.2)	95.5 (7.8)	98.4 (7.9)	96.6 (8.0)	94.8 (6.9)	103.3 (7.4)
	Waist–hip ratio	0.9 (0.1)	0.9 (0.1)	0.9 (0.1)	0.9 (0.1)	0.9 (0.1)	0.9 (0.1)
	Upper arm circumference, cm	26.8 (4.5)	27.6 (5.0)	27.5 (4.7)	27.2 (4.7)	26.2 (4.7)	30.9 (4.9)
	Triceps skin fold, mm	16.1 (7.6)	17.0 (8.1)	15.7 (8.1)	16.2 (9.0)	15.1 (7.2)	21.9 (9.1)
**Blood pressure**							
	Systolic blood pressure, mmHg	120.7 (17.6)	127.6 (19.3)	135.3 (17.5)	133.5 (17.6)	132.8 (18.6)	133.2 (17.3)
	Diastolic blood pressure, mmHg	78.2 (10.9)	81.7 (11.0)	84.7 (11.2)	86.1 (12.9)	82.1 (10.6)	86.0 (11.0)
	Use of blood pressure-lowering medication	201 (6%)	362 (13%)	11 (19%)	17 (24%)	48 (18%)	38 (23%)
**Kidney function**							
	Serum creatinine, mg/dl	1.0 (0.3)	1.0 (0.2)	1.1 (0.2)	1.0 (0.2)	1.0 (0.4)	1.0 (0.2)
	CKD-EPI-Scr	81.0 (16.7)	77.1 (16.1)	69.4 (15.7)	79.9 (21.1)	70.0 (16.2)	79.2 (16.0)
**Lipid profile**							
	Triglycerides, mg/dl	121.4 (90.0)	153.6 (120.2)	233.2 (161.8)	283.3 (316.9)	190.2 (164.4)	292.8 (253.2)
	Total Cholesterol, mg/dl	180.9 (35.7)	194.9 (39.0)	197.9 (36.2)	211.2 (50.2)	204.2 (38.7)	213.6 (45.0)
	High-density lipoprotein, mg/dl	56.7 (17.1)	55.6 (18.0)	49.8 (11.9)	48.2 (13.6)	55.6 (15.5)	47.7 (13.5)
	Low-density lipoprotein, mg/dl	110.4 (34.2)	122.3 (39.4)	117.6 (36.4)	124.0 (42.5)	122.9 (42.4)	124.8 (61.7)
	Apolipoprotein A-1, g/L	1.2 (0.4)	1.1 (0.4)	1.1 (0.3)	1.1 (0.4)	1.2 (0.3)	1.0 (0.3)
	Apolipoprotein B, g/L	0.9 (0.3)	1.0 (0.3)	1.0 (0.3)	1.1 (0.3)	1.0 (0.3)	1.1 (0.3)
	Lipoprotein (a), g/L	0.08 (0.04, 0.17)	0.08 (0.04, 0.17)	0.08 (0.05, 0.17)	0.07 (0.04, 0.14)	0.09 (0.04, 0.17)	0.07 (0.03, 0.15)

Values are mean (SD) for continuous variables and number (percentage) for categorical variables.

SIRD, severe insulin-resistant diabetes; SIDD, severe insulin-deficient diabetes; MARD, mild age-related diabetes; MOD, mild obesity-related diabetes; eGFR, estimated glomerular filtration rate; CKD-EPI-Scr, Chronic Kidney Disease Epidemiology Collaboration, serum creatinine.

Significant differences were found between glycemic statuses for different lipid indices, with the largest effect sizes in the SIDD and MOD sub-phenotypes (**Figure 2B**). Specifically, compared to participants with normoglycemia, those in other subgroups had significantly higher levels of the standardized triglyceride, with mean difference (95% CI) 0.26 (0.21, 0.31) in prediabetes, 0.55 (0.43, 0.67) in MARD, 0.89 (0.64, 1.14) in SIRD, 1.28 (1.06, 1.50) in SIDD, and 1.35 (1.20, 1.50) in MOD in the full models (**Table S5**). By comparing the effect sizes among glycemic statuses and different lipid outcomes, we found that triglyceride was most distinct among the four T2D sub-phenotypes. In contrast, the distribution among T2D sub-phenotypes was relatively the same for HDL and LDL. Kidney function assessed by eGFR had opposite effect directions for different T2D sub-phenotypes (**Figure 2B**). Compared to individuals with normoglycemia, those with prediabetes [0.09 (0.05, 0.13)], SIDD [0.29 (0.11, 0.46)], and MOD [0.17 (0.05, 0.28)] had significantly higher eGFR values, while lower eGFR values were reported in the SIRD group [−0.20 (−0.39, 0.00)] in the full models (**Table S5**).

In total, by comparing the effect sizes for all outcomes among different glycemic statuses, we found that SIRD, SIDD, and MOD had higher cardiovascular abnormalities. In contrast, the risk effect sizes among people in the MARD sub-phenotype were similar to those in prediabetes.

### Trajectories of Cardiovascular Risk Factors Before Diabetes Diagnosis

**Figure 3:**
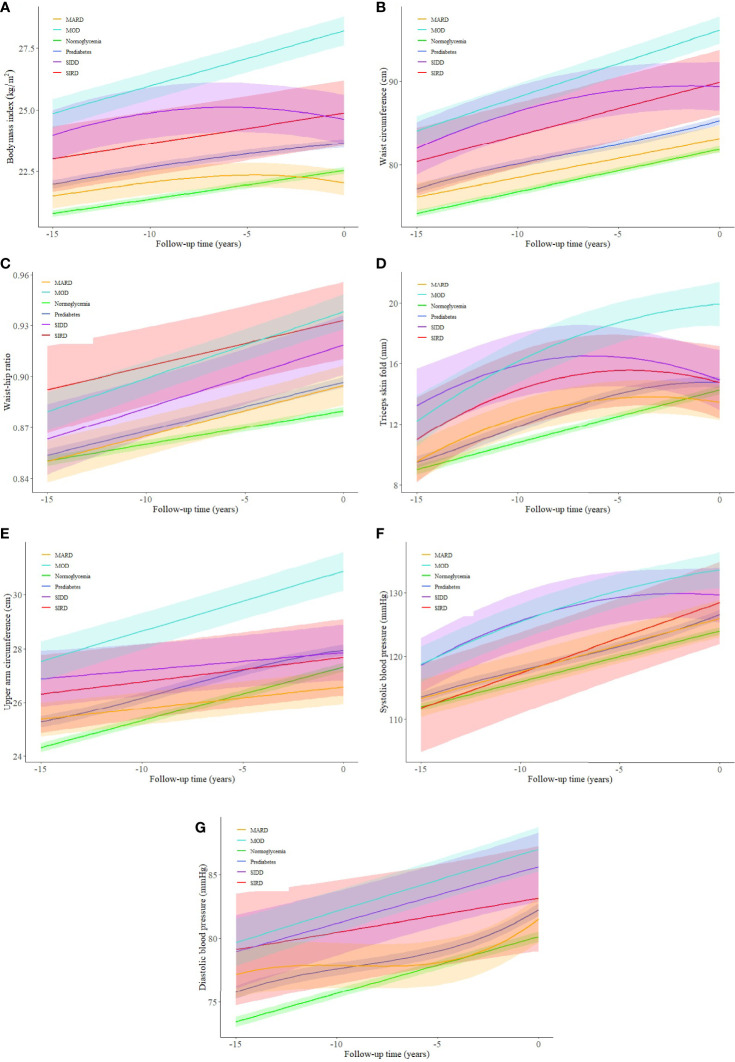
**(A–G)** Trajectories of cardiovascular risk factors for a hypothetical 50-year-old man (in year 0) from 15 years before type 2 diabetes diagnosis among different glycemic statuses. SIRD, severe insulin-resistant diabetes; SIDD, severe insulin-deficient diabetes; MARD, mild age-related diabetes; MOD, mild obesity-related diabetes. The shaded areas represent the 95% confident intervals around the trajectories.

Trajectories of BMI between different glycemic statuses were significantly different (*p* < 0.003), except for the pairwise comparisons between normoglycemia and MARD (*p* = 0.059), and between SIRD and SIDD (**Figure 3A**, *p* = 0.882). The MARD sub-phenotype had a BMI level even lower than that of prediabetes and declined gradually almost 5 years before the T2D diagnosis. Compared to other subgroups, the MOD sub-phenotype had a significantly higher burden of obesity, with the BMI level reaching the overweight threshold (25 kg/m^2^) 15 years before diabetes diagnosis and increasing stably throughout follow-up. Trajectories of waist circumference also showed similar trends, with significant differences found among pairwise comparisons except for the differences between SIRD and SIDD (**Figure 3B**, *p* = 0.556). Compared to other subgroups, the MOD sub-phenotype had a higher level of waist circumference and a larger slope. Trajectories of waist–hip ratio were less distinct. Although normoglycemia, prediabetes, and MOD sub-phenotype had lower levels of waist–hip ratio, no significant difference was found between SIRD, SIDD, and MOD (**Figure 3C**, *p* = 0.821 for SIRD *vs.* SIDD; *p* = 0.069 for SIRD *vs.* MOD; *p* = 0.036 for SIDD *vs.* MOD) during the pairwise comparing. The trajectories of the triceps skinfold and upper arm circumference showed similar changing trends among different glycemic statuses, with the MOD sub-phenotype increasing steadily throughout follow-up (**Figures 3D, E**).

Unlike body fat distribution indices, blood pressure trajectories among T2D sub-phenotypes were less distinct from each other. A steadily increasing blood pressure trend was noticed in all subgroups, with the SIDD and MOD sub-phenotypes having higher average blood pressure levels during follow-up (**Figures 3F, G**). Pairwise comparisons between T2D sub-phenotypes suggested significant differences between SIRD and MARD (*p* < 0.001), and between MOD and MARD (*p* < 0.001) for both systolic and diastolic blood pressure.

## Discussion

Our study supported the application of T2D clustering among the Chinese population, with four T2D sub-phenotypes driven. Chinese people within different T2D sub-phenotypes tended to present different cardiorenal risk profiles. People clustered in the SIRD, SIDD, or MOD had higher cardiorenal risk abnormalities, while for those in the MARD sub-phenotype, the risk burden was similar to those with prediabetes. Up to 10 years before T2D diagnosis, people with the MOD sub-phenotype had a faster increment in BMI, waist, upper arm circumference, and triceps skinfold than the other T2D sub-phenotypes.

The ethnic difference for diabetes has long been discussed. Compared to white individuals, South Asians tend to have a faster increase in FBG, quicker decline in insulin sensitivity, and more marked disturbance in beta-cell compensation before diabetes diagnosis (13). So far, several studies have verified the generalizability of this stratifying approach. Still, the population distribution within diabetes clusters is different among different ethnicities. A prior study reported that people with diabetes born in the Middle East have more SIDD and MOD sub-phenotypes than native Swedes (7). Similar to our results, Zou et al. found a smaller percentage of patients in the SIRD sub-phenotype than the original Swedish study (8.6% *vs.* 16.3%). This could be due to surrogate parameters for the cluster variables and partly attributable to insulin resistance, which is positively associated with age in Western countries but negatively associated with age in the Chinese population (6). Considering different cardiorenal risk profiles and heterogeneous clinical progressions between T2D sub-phenotypes, ethnic differences should be further investigated for their future applications.

By comparing the effect sizes between different body fat distribution indices and lipid components, all T2D sub-phenotypes have different waist, waist–hip ratio, triglyceride, and total cholesterol distributions. This suggests current classifying method has, at least partly, captured their heterogeneities among diabetic patients. Also, compared to BMI, waist circumference shows a similar distribution and equivalent effect sizes among T2D sub-phenotypes, indicating that including both BMI and waist circumference as cluster variables in the study might be information redundancy (23, 24). However, the HDL level was much less distinct among different diabetes sub-phenotypes. HDL has been known as an explanatory variable of metabolic syndrome and insulin resistance (25). A large genomic study has confirmed the causal link between HDL and T2D (26). Therefore, HDL may be considered an additional cluster variable to help further tailor and target high-risk patients.

The utility of T2D sub-phenotypes for identifying diabetes subgroups with a higher risk of future microvascular complications has been reported (5, 7–9). Here we found that the SIRD cluster has much lower eGFR than other diabetes sub-phenotypes. This is worth noting because kidney function has a major impact on prescribing blood pressure-lowering medications for adults with diabetes (11, 27, 28). For patients with diabetes and hypertension, using an angiotensin-converting enzyme inhibitor or an angiotensin-receptor blocker is strongly recommended for those without chronic kidney disease. These drugs can reduce the risk of cardiovascular events and retard the progression of kidney disease. People clustered as SIRD tend to have accelerated macroalbuminuria, diabetic kidney disease, and end-stage kidney disease (5). Repeated assessments have further confirmed this by showing that the SIRD sub-phenotype had decreased eGFR and increased cystatin-C levels, at both baseline and 5-year follow-up, despite good metabolic control (8). In total, these findings suggest the utility of T2D sub-phenotypes for hypertension management among diabetes.

In complement to the former study, which reported that cluster membership changes as the disease progress (8), our results indicate that for MOD, its phenotypic character occurs a long time before the diabetes diagnosis. Therefore, early prevention should be required concerning its high prevalence and adverse cardiovascular risk burden. Similar to the cross-sectional findings, results from the trajectory analysis also showed that the MARD cluster has a very low obesity burden and blood pressure levels before diagnosis, almost equivalent to those in prediabetes. Since nearly half of new-onset T2D belongs to MARD (46.9%), the “one-size-fits-all” diabetes prevention and treatment strategies should be reconsidered.

The strengths of this study include the cohort study design and long follow-up time. We had detailed information regarding possible confounders. Another strength is using both the cluster analysis and trajectory analysis, enabling the investigation of cardiovascular risk factors changes before diagnosis. Taken together, our study filled in a certain knowledge gap about precision medicine for diabetes early prevention. However, our study only included Chinese individuals, limiting our findings’ generalizability to other ethnicities. In addition, according to the original cohort protocol, the participants who left in one survey year may have moved back later, and new participants have been recruited since 1997 as replenishment samples. Therefore, it is complex to determine response rates and attrition in each survey. Also, glycemic traits such as FBG, HbA1c, and insulin were only measured and analyzed cross-sectionally; therefore, we could not study any sub-phenotype transition during follow-up. Studies with the repeated measurements of cluster variables and even omics (genomics and metabolomics) data are needed further to elucidate the pathophysiological mechanism of different T2D sub-phenotypes.

In summary, our study showed that the data-driven diabetes sub-phenotypes were applicable in the Chinese population. The SIRD, SIDD, and MOD sub-phenotypes had worse cardiorenal abnormalities, while the MARD sub-phenotype had a low-risk burden equivalent to prediabetes. Compared with other sub-phenotypes, patients with MOD have a faster increment in obesity indices up to 10 years before diagnosis. Our findings can help improve early prevention and targeted treatment for diabetes.

## Data Availability Statement

Publicly available datasets were analyzed in this study. These data can be found here: https://www.cpc.unc.edu/projects/china.

## Ethics Statement

The studies involving human participants were reviewed and approved by the Institutional Review Committees of the University of North Carolina at Chapel Hill, NC, USA, and the China National Institute of Nutrition and Food Safety at the Chinese Center for Disease Control and Prevention, Beijing, China. The patients/participants provided their written informed consent to participate in this study.

## Author Contributions

HG, KW, and FA are responsible for the study concept and design. HG and KW composed the statistical dataset, performed the statistical analyses, and wrote the manuscript. All authors contributed to the interpretation of the data and critical revision of the manuscript. KW is the guarantor of this work and had full access to all data in the study and takes responsibility for the integrity and the accuracy of the data analysis.

## Conflict of Interest

The authors declare that the research was conducted in the absence of any commercial or financial relationships that could be construed as a potential conflict of interest.

## Publisher’s Note

All claims expressed in this article are solely those of the authors and do not necessarily represent those of their affiliated organizations, or those of the publisher, the editors and the reviewers. Any product that may be evaluated in this article, or claim that may be made by its manufacturer, is not guaranteed or endorsed by the publisher.
