# Supplementary material for: Cardiorenal Risk Profiles Among Data-Driven Type 2 Diabetes Sub-Phenotypes: A Post-Hoc Analysis of the China Health and Nutrition Survey
Source: Front Endocrinol (Lausanne). 2022 Apr 6;13:828403. doi: 10.3389/fendo.2022.828403 (PMC9019482; doi:10.3389/fendo.2022.828403)

Supplementary Material

**Supplementary Table 1.** Characteristics about glycemic traits among different glycemic statuses

|  | **Normoglycemia (n=3436)** | **Prediabetes (n=2735)** | **SIRD (n=57)** | **SIDD (n=72)** | **MARD (n=261)** | **MOD (n=167)** |
| --- | --- | --- | --- | --- | --- | --- |
| Fasting blood glucose, mmol/L | 4.9 (0.4) | 5.5 (0.6) | 7.8 (2.0) | 12.1 (3.5) | 7.2 (1.6) | 7.2 (1.8) |
| Insulin, uU/mL | 10.7 (6.7) | 14.7 (12.3) | 76.4 (38.8) | 15.7 (9.8) | 16.0 (9.8) | 21.9 (13.4) |
| Hemoglobin A1c, % | 5.2 (0.4) | 5.8 (0.4) | 6.1 (0.8) | 10.3 (1.2) | 6.6 (0.8) | 6.7 (0.8) |
| HOMA-IR | 2.3 (1.5) | 3.7 (3.4) | 28.9 (19.1) | 8.7 (7.0) | 5.4 (4.2) | 7.4 (5.8) |
| HOMA-β | 173.9 (122.0) | 158.1 (117.8) | 372.7 (125.7) | 47.5 (46.2) | 93.3 (49.5) | 131.2 (74.6) |

**Note. V**alues are mean (standard deviation) or median (interquartile range) for continuous variables. SIRD: severe insulin-resistant diabetes; SIDD: severe insulin-deficient diabetes; MARD: mild age-related diabetes; MOD: mild obesity-related diabetes; HOMA-IR, homeostatic model assessment of insulin resistance; HOMA-β, Homeostatic model assessment of beta-cell function.

**Supplementary Table 2.** The numbers of participants attended at different waves

| **Wave** | **Numbers of participants among different glycemic statuses** | | | | | | **Total** |
| --- | --- | --- | --- | --- | --- | --- | --- |
|  | **Normoglycemia** | **Prediabetes** | **SIRD** | **SIDD** | **MARD** | **MOD** |  |
| 2009 | 3436 | 2735 | 57 | 72 | 261 | 167 | 6728 |
| 2006 | 2818 | 2313 | 51 | 56 | 228 | 146 | 5612 |
| 2004 | 2542 | 2106 | 41 | 53 | 204 | 135 | 5081 |
| 2000 | 2403 | 1936 | 41 | 47 | 196 | 131 | 4754 |
| 1997 | 2010 | 1688 | 26 | 41 | 167 | 105 | 4037 |
| 1993 | 1865 | 1563 | 34 | 43 | 157 | 97 | 3759 |
| 1991 | 1871 | 1584 | 34 | 44 | 157 | 97 | 3787 |
| 1989 | 1846 | 1575 | 33 | 45 | 158 | 97 | 3754 |

**Supplementary Table 3.** The median follow-up time (years) for different cardiovascular factors in each glycemic status during trajectory analysis

| **Outcomes** | **Median follow-up time (years) in each glycemic spectrum** | | | | | |
| --- | --- | --- | --- | --- | --- | --- |
|  | **Normoglycemia** | **Prediabetes** | **SIRD** | **SIDD** | **MARD** | **MOD** |
| Body mass index | 18 | 18 | 18 | 18 | 18 | 18 |
| Waist circumference | 12 | 12 | 12 | 12 | 12 | 12 |
| Waist-hip ratio | 12 | 12 | 12 | 12 | 12 | 12 |
| Triceps skinfold | 12 | 12 | 12 | 12 | 12 | 12 |
| Upper arm circumference | 16 | 16 | 16 | 16 | 16 | 16 |
| Systolic blood pressure | 16 | 16 | 16 | 16 | 16 | 16 |
| Diastolic blood pressure | 16 | 16 | 16 | 16 | 16 | 16 |

**Supplementary Table 4.** The numbers of observations for different cardiovascular factors in each glycemic status during trajectory analysis

| **Outcomes** | **Numbers of observations in each glycemic spectrum** | | | | | |
| --- | --- | --- | --- | --- | --- | --- |
|  | **Normoglycemia** | **Prediabetes** | **SIRD** | **SIDD** | **MARD** | **MOD** |
| Body mass index | 15850 | 13266 | 259 | 325 | 1333 | 823 |
| Waist circumference | 12890 | 10855 | 216 | 263 | 1106 | 682 |
| Waist-hip ratio | 12806 | 10784 | 213 | 262 | 1099 | 678 |
| Triceps skinfold | 12669 | 10661 | 211 | 258 | 1081 | 674 |
| Upper arm circumference | 15598 | 13059 | 261 | 322 | 1312 | 817 |
| Systolic blood pressure | 14315 | 12127 | 240 | 302 | 1214 | 762 |
| Diastolic blood pressure | 14315 | 12127 | 240 | 302 | 1214 | 762 |

**Supplementary Table 5**. Pairwise comparison of cardiovascular risk factors among glycemic statuses, using normoglycemia as the reference

| **Outcomes** | | **Subgroup** | **Crude Model** | | **Full Model** | |
| --- | --- | --- | --- | --- | --- | --- |
|  |  |  | **Estimate** | ***p*** | **Estimate** | ***p*** |
| **Body fat distribution** | | | | | | |
|  | **BMI** | Normoglycemia | Reference | - | Reference | - |
|  |  | Prediabetes | 0.40 (0.35, 0.45) | <0.001 | 0.38 (0.33, 0.42) | <0.001 |
|  |  | SIRD | 0.89 (0.65, 1.14) | <0.001 | 0.88 (0.63, 1.12) | <0.001 |
|  |  | SIDD | 0.66 (0.44, 0.88) | <0.001 | 0.58 (0.37, 0.80) | <0.001 |
|  |  | MARD | 0.07 (-0.05, 0.19) | 0.23 | 0.04 (-0.07, 0.16) | 0.464 |
|  |  | MOD | 1.81 (1.67, 1.96) | <0.001 | 1.74 (1.60, 1.89) | <0.001 |
|  | **Waist** | Normoglycemia | Reference | - | Reference | - |
|  |  | Prediabetes | 0.39 (0.34, 0.43) | <0.001 | 0.37 (0.32, 0.41) | <0.001 |
|  |  | SIRD | 0.84 (0.59, 1.08) | <0.001 | 0.82 (0.57, 1.06) | <0.001 |
|  |  | SIDD | 0.81 (0.60, 1.03) | <0.001 | 0.75 (0.53, 0.96) | <0.001 |
|  |  | MARD | 0.28 (0.16, 0.40) | <0.001 | 0.25 (0.13, 0.36) | <0.001 |
|  |  | MOD | 1.51 (1.36, 1.65) | <0.001 | 1.44 (1.30, 1.59) | <0.001 |
|  | **WHR** | Normoglycemia | Reference | - | Reference | - |
|  |  | Prediabetes | 0.21 (0.16, 0.26) | <0.001 | 0.20 (0.15, 0.25) | <0.001 |
|  |  | SIRD | 0.45 (0.20, 0.69) | <0.001 | 0.44 (0.19, 0.69) | <0.001 |
|  |  | SIDD | 0.67 (0.45, 0.89) | <0.001 | 0.64 (0.42, 0.86) | <0.001 |
|  |  | MARD | 0.14 (0.02, 0.26) | 0.022 | 0.12 (0.00, 0.24) | 0.047 |
|  |  | MOD | 0.79 (0.64, 0.93) | <0.001 | 0.75 (0.61, 0.90) | <0.001 |
|  | **Upper arm circumference** | Normoglycemia | Reference | - | Reference | - |
|  |  | Prediabetes | 0.22 (0.17, 0.27) | <0.001 | 0.21 (0.16, 0.26) | <0.001 |
|  |  | SIRD | 0.22 (-0.04, 0.47) | 0.099 | 0.22 (-0.03, 0.48) | 0.089 |
|  |  | SIDD | 0.10 (-0.13, 0.32) | 0.404 | 0.06 (-0.17, 0.29) | 0.613 |
|  |  | MARD | -0.02 (-0.15, 0.10) | 0.699 | -0.04 (-0.16, 0.09) | 0.533 |
|  |  | MOD | 0.87 (0.72, 1.02) | <0.001 | 0.83 (0.68, 0.98) | <0.001 |
|  | **Triceps skin fold** | Normoglycemia | Reference | - | Reference | - |
|  |  | Prediabetes | 0.17 (0.12, 0.22) | <0.001 | 0.16 (0.11, 0.21) | <0.001 |
|  |  | SIRD | 0.07 (-0.18, 0.32) | 0.584 | 0.07 (-0.18, 0.32) | 0.598 |
|  |  | SIDD | 0.12 (-0.10, 0.34) | 0.299 | 0.08 (-0.14, 0.30) | 0.481 |
|  |  | MARD | -0.01 (-0.13, 0.11) | 0.881 | -0.03 (-0.15, 0.10) | 0.68 |
|  |  | MOD | 0.80 (0.65, 0.95) | <0.001 | 0.76 (0.61, 0.91) | <0.001 |
| **Blood pressure** | | | | | | |
|  | **SBP** | Normoglycemia | Reference | - | Reference | - |
|  |  | Prediabetes | 0.13 (0.08, 0.17) | <0.001 | 0.12 (0.08, 0.17) | <0.001 |
|  |  | SIRD | 0.37 (0.15, 0.59) | 0.001 | 0.37 (0.15, 0.59) | 0.001 |
|  |  | SIDD | 0.33 (0.13, 0.53) | 0.001 | 0.33 (0.13, 0.53) | 0.001 |
|  |  | MARD | 0.17 (0.06, 0.28) | 0.002 | 0.16 (0.05, 0.27) | 0.003 |
|  |  | MOD | 0.36 (0.23, 0.49) | <0.001 | 0.35 (0.22, 0.49) | <0.001 |
|  | **DBP** | Normoglycemia | Reference | - | Reference | - |
|  |  | Prediabetes | 0.18 (0.13, 0.23) | <0.001 | 0.18 (0.13, 0.22) | <0.001 |
|  |  | SIRD | 0.36 (0.11, 0.60) | 0.004 | 0.37 (0.13, 0.61) | 0.003 |
|  |  | SIDD | 0.46 (0.24, 0.67) | <0.001 | 0.46 (0.24, 0.67) | <0.001 |
|  |  | MARD | 0.11 (-0.01, 0.23) | 0.067 | 0.10 (-0.02, 0.22) | 0.096 |
|  |  | MOD | 0.48 (0.33, 0.62) | <0.001 | 0.47 (0.33, 0.61) | <0.001 |
| **Lipid profile** | | | | | | |
|  | **TG** | Normoglycemia | Reference | - | Reference | - |
|  |  | Prediabetes | 0.27 (0.22, 0.32) | <0.001 | 0.26 (0.21, 0.31) | <0.001 |
|  |  | SIRD | 0.92 (0.67, 1.17) | <0.001 | 0.89 (0.64, 1.14) | <0.001 |
|  |  | SIDD | 1.31 (1.08, 1.53) | <0.001 | 1.28 (1.06, 1.50) | <0.001 |
|  |  | MARD | 0.58 (0.45, 0.70) | <0.001 | 0.55 (0.43, 0.67) | <0.001 |
|  |  | MOD | 1.39 (1.24, 1.54) | <0.001 | 1.35 (1.20, 1.50) | <0.001 |
|  | **TC** | Normoglycemia | Reference | - | Reference | - |
|  |  | Prediabetes | 0.27 (0.22, 0.32) | <0.001 | 0.26 (0.21, 0.31) | <0.001 |
|  |  | SIRD | 0.30 (0.05, 0.55) | 0.018 | 0.32 (0.07, 0.57) | 0.011 |
|  |  | SIDD | 0.70 (0.48, 0.92) | <0.001 | 0.70 (0.47, 0.92) | <0.001 |
|  |  | MARD | 0.41 (0.29, 0.53) | <0.001 | 0.40 (0.28, 0.52) | <0.001 |
|  |  | MOD | 0.78 (0.63, 0.93) | <0.001 | 0.76 (0.61, 0.91) | <0.001 |
|  | **HDL** | Normoglycemia | Reference | - | Reference | - |
|  |  | Prediabetes | -0.11 (-0.16, -0.05) | <0.001 | -0.10 (-0.15, -0.05) | <0.001 |
|  |  | SIRD | -0.46 (-0.72, -0.20) | <0.001 | -0.43 (-0.69, -0.17) | 0.001 |
|  |  | SIDD | -0.51 (-0.74, -0.28) | <0.001 | -0.48 (-0.71, -0.26) | <0.001 |
|  |  | MARD | -0.15 (-0.28, -0.02) | 0.021 | -0.16 (-0.28, -0.03) | 0.015 |
|  |  | MOD | -0.55 (-0.70, -0.39) | <0.001 | -0.55 (-0.71, -0.40) | <0.001 |
|  | **LDL** | Normoglycemia | Reference | - | Reference | - |
|  |  | Prediabetes | 0.22 (0.17, 0.27) | <0.001 | 0.22 (0.17, 0.27) | <0.001 |
|  |  | SIRD | 0.05 (-0.20, 0.30) | 0.674 | 0.07 (-0.18, 0.33) | 0.566 |
|  |  | SIDD | 0.28 (0.05, 0.51) | 0.016 | 0.27 (0.05, 0.50) | 0.018 |
|  |  | MARD | 0.14 (0.01, 0.26) | 0.028 | 0.14 (0.02, 0.26) | 0.027 |
|  |  | MOD | 0.31 (0.16, 0.46) | <0.001 | 0.31 (0.16, 0.46) | <0.001 |
|  | **Apo-A** | Normoglycemia | Reference | - | Reference | - |
|  |  | Prediabetes | -0.15 (-0.20, -0.10) | <0.001 | -0.14 (-0.20, -0.09) | <0.001 |
|  |  | SIRD | -0.31 (-0.57, -0.05) | 0.021 | -0.28 (-0.54, -0.02) | 0.035 |
|  |  | SIDD | -0.16 (-0.39, 0.07) | 0.167 | -0.13 (-0.36, 0.10) | 0.269 |
|  |  | MARD | -0.18 (-0.31, -0.06) | 0.005 | -0.18 (-0.31, -0.06) | 0.005 |
|  |  | MOD | -0.37 (-0.53, -0.22) | <0.001 | -0.37 (-0.53, -0.22) | <0.001 |
|  | **Apo-B** | Normoglycemia | Reference | - | Reference | - |
|  |  | Prediabetes | 0.27 (0.22, 0.32) | <0.001 | 0.26 (0.21, 0.31) | <0.001 |
|  |  | SIRD | 0.41 (0.16, 0.66) | 0.001 | 0.43 (0.18, 0.68) | 0.001 |
|  |  | SIDD | 0.76 (0.54, 0.98) | <0.001 | 0.74 (0.52, 0.97) | <0.001 |
|  |  | MARD | 0.33 (0.20, 0.45) | <0.001 | 0.31 (0.19, 0.44) | <0.001 |
|  |  | MOD | 0.68 (0.53, 0.83) | <0.001 | 0.66 (0.51, 0.81) | <0.001 |
|  | **Lip-a** | Normoglycemia | Reference | - | Reference | - |
|  |  | Prediabetes | -0.03 (-0.09, 0.02) | 0.184 | -0.04 (-0.09, 0.01) | 0.138 |
|  |  | SIRD | 0.10 (-0.16, 0.36) | 0.453 | 0.10 (-0.16, 0.36) | 0.461 |
|  |  | SIDD | 0.02 (-0.21, 0.25) | 0.87 | 0.01 (-0.22, 0.25) | 0.908 |
|  |  | MARD | -0.04 (-0.16, 0.09) | 0.572 | -0.05 (-0.18, 0.08) | 0.467 |
|  |  | MOD | -0.17 (-0.32, -0.01) | 0.033 | -0.18 (-0.33, -0.02) | 0.025 |
| **Kidney function** | | | | | | |
|  | **eGFR** | Normoglycemia | Reference | - | Reference | - |
|  |  | Prediabetes | 0.09 (0.05, 0.12) | <0.001 | 0.09 (0.05, 0.13) | <0.001 |
|  |  | SIRD | -0.19 (-0.39, 0.00) | 0.05 | -0.20 (-0.39, 0.00) | 0.046 |
|  |  | SIDD | -0.25 (-0.08, 0.43) | 0.004 | 0.29 (0.11, 0.46) | 0.001 |
|  |  | MARD | 0.02 (-0.08, 0.11) | 0.713 | 0.02 (-0.08, 0.11) | 0.709 |
|  |  | MOD | -0.15 (-0.04, 0.27) | 0.009 | 0.17 (0.05, 0.28) | 0.004 |

**Note.** SIRD: severe insulin-resistant diabetes; SIDD: severe insulin-deficient diabetes; MARD: mild age-related diabetes; MOD: mild obesity-related diabetes.

Crude model adjusted for age, sex, education, residence, marital status.

Full Model additionally adjusted for smoke status, alcohol consumption, sleep, excise status, sedentary behavior, blood pressure-lowering medication, and prevalent cardiopulmonary disease (myocardial infarction, stroke, and asthma).

For blood pressure outcomes, the use of blood pressure-lowering medication was also included in the crude model.

**Supplementary Table 6**. P-value for pairwise comparison of cardiovascular risk factors among glycemic statuses

| **Outcomes** | | **Cluster comparison** | | **P-value for crude model** | **P-value for full model** |
| --- | --- | --- | --- | --- | --- |
| **Body fat distribution** | | | | | |
|  | **BMI** | Prediabetes | SIRD | <0.001 | <0.001 |
|  |  | Prediabetes | SIDD | 0.017 | 0.053 |
|  |  | Prediabetes | MARD | <0.001 | <0.001 |
|  |  | Prediabetes | MOD | <0.001 | <0.001 |
|  |  | SIRD | SIDD | 0.177 | 0.104 |
|  |  | SIRD | MARD | <0.001 | <0.001 |
|  |  | SIRD | MOD | <0.001 | <0.001 |
|  |  | SIDD | MARD | <0.001 | <0.001 |
|  |  | SIDD | MOD | <0.001 | <0.001 |
|  |  | MARD | MOD | <0.001 | <0.001 |
|  | **Waist** | Prediabetes | SIRD | <0.001 | <0.001 |
|  |  | Prediabetes | SIDD | <0.001 | <0.001 |
|  |  | Prediabetes | MARD | 0.135 | 0.104 |
|  |  | Prediabetes | MOD | <0.001 | <0.001 |
|  |  | SIRD | SIDD | 0.951 | 0.863 |
|  |  | SIRD | MARD | <0.001 | <0.001 |
|  |  | SIRD | MOD | <0.001 | <0.001 |
|  |  | SIDD | MARD | <0.001 | <0.001 |
|  |  | SIDD | MOD | <0.001 | <0.001 |
|  |  | MARD | MOD | <0.001 | <0.001 |
|  | **WHR** | Prediabetes | SIRD | 0.048 | 0.039 |
|  |  | Prediabetes | SIDD | <0.001 | <0.001 |
|  |  | Prediabetes | MARD | 0.481 | 0.442 |
|  |  | Prediabetes | MOD | <0.001 | <0.001 |
|  |  | SIRD | SIDD | 0.134 | 0.241 |
|  |  | SIRD | MARD | 0.029 | 0.024 |
|  |  | SIRD | MOD | 0.013 | 0.031 |
|  |  | SIDD | MARD | <0.001 | <0.001 |
|  |  | SIDD | MOD | 0.384 | 0.354 |
|  |  | MARD | MOD | <0.001 | <0.001 |
|  | **Upper arm circumference** | Prediabetes | SIRD | 0.96 | 0.801 |
|  |  | Prediabetes | SIDD | 0.377 | 0.27 |
|  |  | Prediabetes | MARD | <0.001 | <0.001 |
|  |  | Prediabetes | MOD | <0.001 | <0.001 |
|  |  | SIRD | SIDD | 0.586 | 0.459 |
|  |  | SIRD | MARD | 0.069 | 0.07 |
|  |  | SIRD | MOD | <0.001 | <0.001 |
|  |  | SIDD | MARD | 0.194 | 0.284 |
|  |  | SIDD | MOD | <0.001 | <0.001 |
|  |  | MARD | MOD | <0.001 | <0.001 |
|  | **Triceps skin fold** | Prediabetes | SIRD | 0.448 | 0.503 |
|  |  | Prediabetes | SIDD | 0.64 | 0.509 |
|  |  | Prediabetes | MARD | 0.004 | 0.005 |
|  |  | Prediabetes | MOD | <0.001 | <0.001 |
|  |  | SIRD | SIDD | 0.756 | 0.602 |
|  |  | SIRD | MARD | 0.503 | 0.729 |
|  |  | SIRD | MOD | <0.001 | <0.001 |
|  |  | SIDD | MARD | 0.257 | 0.312 |
|  |  | SIDD | MOD | <0.001 | <0.001 |
|  |  | MARD | MOD | <0.001 | <0.001 |
| **Blood pressure** | | | | | |
|  | **SBP** | Prediabetes | SIRD | 0.036 | 0.033 |
|  |  | Prediabetes | SIDD | 0.042 | 0.036 |
|  |  | Prediabetes | MARD | 0.459 | 0.557 |
|  |  | Prediabetes | MOD | <0.001 | <0.001 |
|  |  | SIRD | SIDD | 0.782 | 0.649 |
|  |  | SIRD | MARD | 0.118 | 0.092 |
|  |  | SIRD | MOD | 0.905 | 0.859 |
|  |  | SIDD | MARD | 0.165 | 0.191 |
|  |  | SIDD | MOD | 0.796 | 0.709 |
|  |  | MARD | MOD | 0.022 | 0.022 |
|  | **DBP** | Prediabetes | SIRD | 0.122 | 0.092 |
|  |  | Prediabetes | SIDD | 0.009 | 0.008 |
|  |  | Prediabetes | MARD | 0.475 | 0.41 |
|  |  | Prediabetes | MOD | <0.001 | <0.001 |
|  |  | SIRD | SIDD | 0.631 | 0.641 |
|  |  | SIRD | MARD | 0.127 | 0.098 |
|  |  | SIRD | MOD | 0.566 | 0.527 |
|  |  | SIDD | MARD | 0.022 | 0.013 |
|  |  | SIDD | MOD | 0.977 | 0.945 |
|  |  | MARD | MOD | <0.001 | <0.001 |
| **Lipid profile** | | | | | |
|  | **TG** | Prediabetes | SIRD | <0.001 | <0.001 |
|  |  | Prediabetes | SIDD | <0.001 | <0.001 |
|  |  | Prediabetes | MARD | <0.001 | <0.001 |
|  |  | Prediabetes | MOD | <0.001 | <0.001 |
|  |  | SIRD | SIDD | 0.336 | 0.316 |
|  |  | SIRD | MARD | 0.412 | 0.455 |
|  |  | SIRD | MOD | 0.199 | 0.221 |
|  |  | SIDD | MARD | 0.042 | 0.047 |
|  |  | SIDD | MOD | 0.846 | 0.933 |
|  |  | MARD | MOD | <0.001 | <0.001 |
|  | **TC** | Prediabetes | SIRD | 0.781 | 0.619 |
|  |  | Prediabetes | SIDD | <0.001 | <0.001 |
|  |  | Prediabetes | MARD | 0.028 | 0.036 |
|  |  | Prediabetes | MOD | <0.001 | <0.001 |
|  |  | SIRD | SIDD | 0.054 | 0.108 |
|  |  | SIRD | MARD | 0.281 | 0.474 |
|  |  | SIRD | MOD | 0.014 | 0.032 |
|  |  | SIDD | MARD | 0.173 | 0.185 |
|  |  | SIDD | MOD | 0.782 | 0.688 |
|  |  | MARD | MOD | 0.019 | 0.01 |
|  | **HDL** | Prediabetes | SIRD | 0.006 | 0.01 |
|  |  | Prediabetes | SIDD | <0.001 | 0.001 |
|  |  | Prediabetes | MARD | 0.355 | 0.272 |
|  |  | Prediabetes | MOD | <0.001 | <0.001 |
|  |  | SIRD | SIDD | 0.746 | 0.863 |
|  |  | SIRD | MARD | 0.006 | 0.012 |
|  |  | SIRD | MOD | 0.477 | 0.407 |
|  |  | SIDD | MARD | <0.001 | 0.003 |
|  |  | SIDD | MOD | 0.71 | 0.482 |
|  |  | MARD | MOD | <0.001 | <0.001 |
|  | **LDL** | Prediabetes | SIRD | 0.233 | 0.304 |
|  |  | Prediabetes | SIDD | 0.588 | 0.561 |
|  |  | Prediabetes | MARD | 0.17 | 0.181 |
|  |  | Prediabetes | MOD | 0.246 | 0.218 |
|  |  | SIRD | SIDD | 0.28 | 0.485 |
|  |  | SIRD | MARD | 0.702 | 0.938 |
|  |  | SIRD | MOD | 0.193 | 0.287 |
|  |  | SIDD | MARD | 0.286 | 0.369 |
|  |  | SIDD | MOD | 0.939 | 0.73 |
|  |  | MARD | MOD | 0.148 | 0.148 |
|  | **Apo-A** | Prediabetes | SIRD | 0.192 | 0.259 |
|  |  | Prediabetes | SIDD | 0.886 | 0.967 |
|  |  | Prediabetes | MARD | 0.635 | 0.569 |
|  |  | Prediabetes | MOD | 0.002 | 0.001 |
|  |  | SIRD | SIDD | 0.349 | 0.261 |
|  |  | SIRD | MARD | 0.152 | 0.138 |
|  |  | SIRD | MOD | 0.382 | 0.45 |
|  |  | SIDD | MARD | 0.78 | 0.977 |
|  |  | SIDD | MOD | 0.036 | 0.027 |
|  |  | MARD | MOD | <0.001 | 0.001 |
|  | **Apo-B** | Prediabetes | SIRD | 0.262 | 0.194 |
|  |  | Prediabetes | SIDD | <0.001 | <0.001 |
|  |  | Prediabetes | MARD | 0.389 | 0.43 |
|  |  | Prediabetes | MOD | <0.001 | <0.001 |
|  |  | SIRD | SIDD | 0.081 | 0.181 |
|  |  | SIRD | MARD | 0.667 | 0.493 |
|  |  | SIRD | MOD | 0.185 | 0.268 |
|  |  | SIDD | MARD | 0.005 | 0.01 |
|  |  | SIDD | MOD | 0.481 | 0.708 |
|  |  | MARD | MOD | <0.001 | 0.004 |
|  | **Lip-a** | Prediabetes | SIRD | 0.331 | 0.331 |
|  |  | Prediabetes | SIDD | 0.653 | 0.667 |
|  |  | Prediabetes | MARD | 0.908 | 0.822 |
|  |  | Prediabetes | MOD | 0.109 | 0.086 |
|  |  | SIRD | SIDD | 0.635 | 0.514 |
|  |  | SIRD | MARD | 0.357 | 0.391 |
|  |  | SIRD | MOD | 0.073 | 0.077 |
|  |  | SIDD | MARD | 0.6 | 0.851 |
|  |  | SIDD | MOD | 0.148 | 0.238 |
|  |  | MARD | MOD | 0.18 | 0.131 |
| **Kidney function** | | | | | |
|  | **eGFR** | Prediabetes | SIRD | 0.004 | 0.003 |
|  |  | Prediabetes | SIDD | 0.055 | 0.024 |
|  |  | Prediabetes | MARD | 0.239 | 0.204 |
|  |  | Prediabetes | MOD | 0.268 | 0.158 |
|  |  | SIRD | SIDD | 0.002 | <0.001 |
|  |  | SIRD | MARD | 0.032 | 0.012 |
|  |  | SIRD | MOD | 0.005 | <0.001 |
|  |  | SIDD | MARD | 0.106 | 0.052 |
|  |  | SIDD | MOD | 0.356 | 0.296 |
|  |  | MARD | MOD | 0.228 | 0.125 |

**Note.** SIRD: severe insulin-resistant diabetes; SIDD: severe insulin-deficient diabetes; MARD: mild age-related diabetes; MOD: mild obesity-related diabetes.

Crude model adjusted for age, sex, education, residence, marital status.

Full Model additionally adjusted for smoke status, alcohol consumption, sleep, excise status, sedentary behavior, use of blood pressure-lowering medication, and prevalent cardiopulmonary disease (myocardial infarction, stroke, and asthma).

For blood pressure outcomes, the use of blood pressure-lowering medication was also included in the crude model.

**Supplementary Table 7.** Comparison of characteristics between participants included and excluded due to the missing of exposure or outcome variables

|  | | **Included (n=6728)** | **Excluded (n=1524)** |
| --- | --- | --- | --- |
| Age, years | | 50.9 (15.0) | 48.8 (14.5) |
| Sex, female | | 3616 (53.7%) | 766 (50.3%) |
| Education level | |  |  |
|  | Illiterate | 1638 (24.3%) | 313 (21.0%) |
|  | Primary school | 1315 (19.5%) | 304 (20.4%) |
|  | Middle school | 2236 (33.2%) | 469 (31.5%) |
|  | High school or higher | 1539 (22.9%) | 402 (27.0%) |
| Residence | |  |  |
|  | Urban | 2096 (31.2%) | 561 (37.5%) |
|  | Rural | 4632 (68.8%) | 934 (62.5%) |
| Married | | 5679 (84.4%) | 1272 (84.7%) |
| Current smoker | | 1873 (27.8%) | 427 (28.5%) |
| Frequency of alcohol consumption | |  |  |
|  | No alcohol consumption | 4517 (67.1%) | 983 (67.9%) |
|  | 1-2 times / week or less | 1280 (19.0%) | 282 (19.5%) |
|  | 3-4 times / week or more | 931 (13.8%) | 182 (12.6%) |
| Sleep, hours per day | | 7.8 (2.1) | 7.6 (2.4) |
| Insufficient physical activity | | 6518 (96.9%) | 1444 (98.4%) |
| Sedentary behavior, hours per day | | 0.8 (0.6) | 0.8 (0.6) |
| Prevalent myocardial infarction | | 49 (0.7%) | 20 (1.3%) |
| Prevalent stroke | | 87 (1.3%) | 18 (1.2%) |
| Prevalent asthma | | 82 (1.2%) | 14 (0.9%) |
| **Body fat distribution** | |  |  |
|  | Height, cm | 160.6 (8.6) | 163.3 (8.4) |
|  | Weight, kg | 60.2 (11.1) | 62.8 (11.8) |
|  | Body mass index, kg/m2 | 23.3 (3.4) | 23.5 (3.6) |
|  | Waist circumference, cm | 82.5 (10.2) | 82.5 (10.8) |
|  | Hip circumference, cm | 94.2 (7.7) | 95.0 (8.6) |
|  | Waist-hip ratio | 0.9 (0.1) | 0.9 (0.1) |
|  | Upper arm circumference, cm | 27.2 (4.8) | 27.3 (4.3) |
|  | Triceps skin fold, mm | 16.6 (7.9) | 15.7 (6.6) |
| **Blood pressure** | |  |  |
|  | Systolic blood pressure, mmHg | 124.5 (18.8) | 125.3 (17.8) |
|  | Diastolic blood pressure, mmHg | 80.1 (11.2) | 81.8 (10.7) |
|  | Use of blood pressure-lowering medication | 677 (10.1%) | 109 (7.2%) |
| **Kidney function** | |  |  |
|  | Serum creatinine, mg/dL | 1.0 (0.2) | 1.0 (0.3) |
|  | eGFR (CKD-EPI-Scr) | 78.8 (16.7) | 82.2 (16.3) |
| **Glycemic metabolism** | |  |  |
|  | Fasting blood glucose, mmol/L | 5.4 (1.2) | 5.1 (1.3) |
|  | Insulin, uU/mL | 13.4 (12.1) | 15.2 (37.3) |
|  | Hemoglobin A1c, % | 5.6 (0.8) | 5.5 (0.7) |
|  | HOMA-IR | 3.4 (4.2) | 3.6 (9.4) |
|  | HOMA-β | 163.6 (120.1) | 167.3 (128.2) |
| **Lipid profile** | |  |  |
|  | Triglycerides, mg/dL | 144.1 (123.0) | 156.8 (151.6) |
|  | Total Cholesterol, mg/dL | 188.8 (38.6) | 183.8 (39.0) |
|  | High-density lipoprotein, mg/dL | 55.8 (17.3) | 54.8 (20.2) |
|  | Low-density lipoprotein, mg/dL | 116.3 (38.2) | 110.2 (36.3) |
|  | Apolipoprotein A-1, g/L | 1.2 (0.4) | 1.1 (0.3) |
|  | Apolipoprotein B, g/L | 0.9 (0.3) | 0.9 (0.3) |
|  | Lipoprotein (a), g/L | 0.2 (0.2) | 0.1 (0.2) |

**Note.** Values are mean (standard deviation) or median (interquartile range) for continuous variables and number (percentage) for categorical variables. SIRD: severe insulin-resistant diabetes; SIDD: severe insulin-deficient diabetes; MARD: mild age-related diabetes; MOD: mild obesity-related diabetes; eGFR, estimated glomerular filtration rate; HOMA-IR, homeostatic model assessment of insulin resistance; HOMA-β, Homeostatic model assessment of beta-cell function.

**Supplementary Figure 1**. Flowchart of the study population


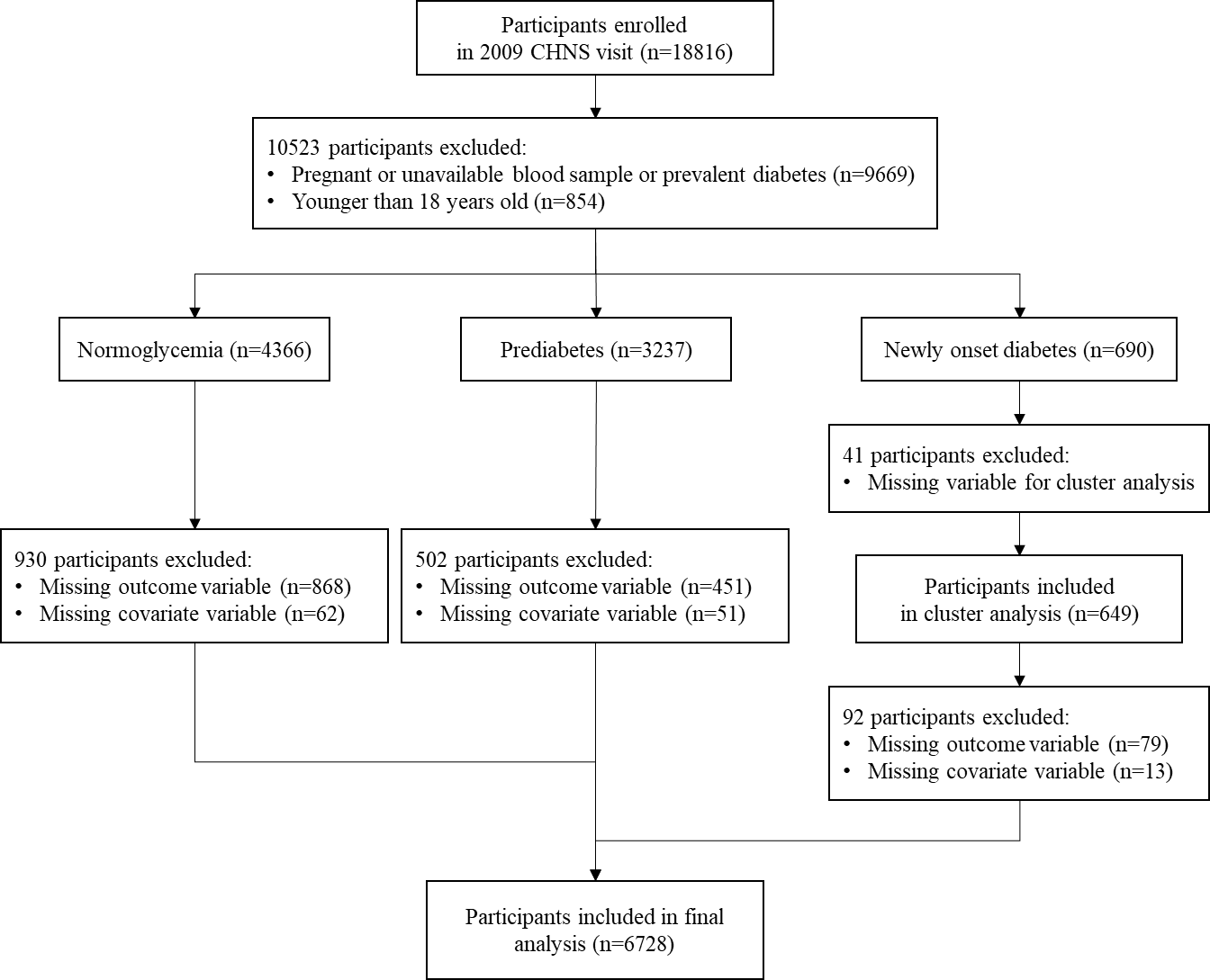

Supplement: Supplementary file 1 [file DataSheet_1.docx]
